# Supplementary material for: Predation and fragmentation portrayed in the statistical structure of prey time series
Source: BMC Ecol. 2009 May 6;9:10. doi: 10.1186/1472-6785-9-10 (PMC2689204; doi:10.1186/1472-6785-9-10)
Supplement: Additional file 2 — Voles and related classes ODDox Documentation. ODDox documentation of the agent-based model (ALMaSS) applied by Hendrichsen et al. The documentation is started by activating main.html. [file 1472-6785-9-10-S2.zip › Vole_ODDox/globals_0x77.html]

ALMaSS ODDox: Class Members

- Main Page
- Related Pages
- Classes
- Files

- File List
- File Members

- All
- Functions
- Variables
- Typedefs
- Enumerations
- Enumerator
- Defines

- \_
- a
- b
- c
- d
- f
- g
- h
- i
- l
- m
- n
- o
- p
- r
- s
- t
- u
- v
- w

Here is a list of all file members with links to the files they belong to:

### - w -

- water
  : treatment.h- WeanedAge
    : Vole\_all.cpp- WeanedWeight
      : Vole\_all.cpp- WEASEL
        : Predators.cpp- weasel\_breed\_day
          : Predators.cpp- weasel\_breed\_threshold
            : Predators.cpp- weasel\_death\_threshold
              : Predators.cpp- weasel\_StartingNo
                : Predators.cpp- winter\_plough
                  : treatment.h- WINTERWHEAT\_BASE
                    : WinterWheat.H- WinterWheatToDo
                      : WinterWheat.H- ww\_autumn\_harrow
                        : WinterWheat.H- WW\_AUTUMN\_PLOUGH
                          : WinterWheat.H- ww\_autumn\_plough
                            : WinterWheat.H- ww\_autumn\_roll
                              : WinterWheat.H- ww\_autumn\_sow
                                : WinterWheat.H- ww\_ferti\_p1
                                  : WinterWheat.H- ww\_ferti\_p2
                                    : WinterWheat.H- ww\_ferti\_p3
                                      : WinterWheat.H- ww\_ferti\_p4
                                        : WinterWheat.H- ww\_ferti\_p5
                                          : WinterWheat.H- ww\_ferti\_s1
                                            : WinterWheat.H- ww\_ferti\_s2
                                              : WinterWheat.H- ww\_ferti\_s3
                                                : WinterWheat.H- ww\_ferti\_s4
                                                  : WinterWheat.H- ww\_ferti\_s5
                                                    : WinterWheat.H- ww\_fungicide
                                                      : WinterWheat.H- ww\_fungicide2
                                                        : WinterWheat.H- ww\_GR
                                                          : WinterWheat.H- ww\_grubning
                                                            : WinterWheat.H- ww\_harvest
                                                              : WinterWheat.H- ww\_hay\_baling
                                                                : WinterWheat.H- ww\_hay\_turning
                                                                  : WinterWheat.H- ww\_herbicide1
                                                                    : WinterWheat.H- ww\_herbicide2
                                                                      : WinterWheat.H- ww\_insecticide1
                                                                        : WinterWheat.H- ww\_insecticide2
                                                                          : WinterWheat.H- ww\_insecticide3
                                                                            : WinterWheat.H- ww\_sleep\_all\_day
                                                                              : WinterWheat.H- ww\_spring\_roll
                                                                                : WinterWheat.H- ww\_start
                                                                                  : WinterWheat.H- ww\_straw\_chopping
                                                                                    : WinterWheat.H- ww\_strigling1
                                                                                      : WinterWheat.H- ww\_strigling2
                                                                                        : WinterWheat.H- ww\_stubble\_harrow1
                                                                                          : WinterWheat.H- ww\_stubble\_harrow2
                                                                                            : WinterWheat.H- ww\_water1
                                                                                              : WinterWheat.H- ww\_water2
                                                                                                : WinterWheat.H

---

Generated on Thu Jan 22 14:13:48 2009 for ALMaSS ODDox by 
 1.5.6
